# Skin Color Variation in Orang Asli Tribes of Peninsular Malaysia

**DOI:** 10.1371/journal.pone.0042752

**Published:** 2012-08-13

**Authors:** Khai C. Ang, Mee S. Ngu, Katherine P. Reid, Mei S. Teh, Zamzuraida S. Aida, Danny XR. Koh, Arthur Berg, Stephen Oppenheimer, Hood Salleh, Mahani M. Clyde, Badrul M. Md-Zain, Victor A. Canfield, Keith C. Cheng

**Affiliations:** 1 Department of Experimental Pathology & Jake Gittlen Cancer Research Foundation, Penn State College of Medicine, Hershey, Pennsylvania, United States of America; 2 School of Environmental and Natural Resource Sciences, Faculty of Science and Technology, Universiti Kebangsaan Malaysia, Bangi, Selangor, Malaysia; 3 Department of Biostatistics & Bioinformatics, Penn State College of Medicine, Hershey, Pennsylvania, United States of America; 4 Institute of Cognitive and Evolutionary Anthropology, School of Anthropology and Museum Ethnography: University of Oxford, Oxford, United Kingdom; 5 Academic Heritage Museum, Chancellery Building, Universiti Kebangsaan Malaysia, Bangi, Selangor, Malaysia; 6 Department of Pharmacology, Penn State College of Medicine, Hershey, Pennsylvania, United States of America; University of Cambridge, United Kingdom

## Abstract

Pigmentation is a readily scorable and quantitative human phenotype, making it an excellent model for studying multifactorial traits and diseases. Convergent human evolution from the ancestral state, darker skin, towards lighter skin colors involved divergent genetic mechanisms in people of European vs. East Asian ancestry. It is striking that the European mechanisms result in a 10–20-fold increase in skin cancer susceptibility while the East Asian mechanisms do not. Towards the mapping of genes that contribute to East Asian pigmentation there is need for one or more populations that are admixed for ancestral and East Asian ancestry, but with minimal European contribution. This requirement is fulfilled by the Senoi, one of three indigenous tribes of Peninsular Malaysia collectively known as the Orang Asli. The Senoi are thought to be an admixture of the Negrito, an ancestral dark-skinned population representing the second of three Orang Asli tribes, and regional Mongoloid populations of Indo-China such as the Proto-Malay, the third Orang Asli tribe. We have calculated skin reflectance-based melanin indices in 492 Orang Asli, which ranged from 28 (lightest) to 75 (darkest); both extremes were represented in the Senoi. Population averages were 56 for Negrito, 42 for Proto-Malay, and 46 for Senoi. The derived allele frequencies for *SLC24A5* and *SLC45A2* in the Senoi were 0.04 and 0.02, respectively, consistent with greater South Asian than European admixture. Females and individuals with the *A111T* mutation had significantly lighter skin (*p* = 0.001 and 0.0039, respectively). Individuals with these derived alleles were found across the spectrum of skin color, indicating an overriding effect of strong skin lightening alleles of East Asian origin. These results suggest that the Senoi are suitable for mapping East Asian skin color genes.

## Introduction

The genetic basis of variation in human skin color across global populations is still largely a mystery. Although partially delineated for Europeans, skin color remains poorly understood for East Asians [1]. For mixed individuals, we are far from specifying skin color purely from genotype. Modern humans are thought to have retained dark skin in equatorial regions such as central Africa in order to prevent destruction of the essential nutrient, folate, by UV light [2]. Conversely, it is thought that lighter skin color was selected for in northerly latitudes to increase the penetration of solar UV light, which is in turn required for a necessary photoactivation step in the formation of vitamin D, another essential vitamin [2], [3]. Selective pressure resulted in convergent evolution towards lighter skin, once each in the European and East Asian branches of human ancestry, through divergent genetic mechanisms.

The differing mechanisms for light skin in Europeans vs. East Asians are associated with disparate skin cancer susceptibility: incidences of the most lethal skin cancer, melanoma [4], are 10–20 fold greater in Europeans than in Africans [4], [5]. In stark contrast, the lighter skin colors of East Asian populations are associated with almost no increase in melanoma; their incidences are comparable with those of Africans [5]. A molecular understanding of this disparity can only be gained after the alleles involved in skin lightening for both groups are identified.

The genetic basis of East Asian skin color is not explained by skin color alleles identified to date. The most pervasive contributor to European skin color is the *A111T* allele of *SLC24A5 (SLC24A5^A111T^*), which is associated with a single nucleotide polymorphism (SNP) at rs1426654. This mutation from the ancestral state is 100% fixed in the CEU (European) population of HapMap [6] and is associated with the strongest genomic signal for selection – as measured by the size of the region of diminished variation – in European genomes [7]. This discovery was made possible by studies of a zebrafish mutant with a nonsense mutation in the orthologous gene: *golden^b1^* [7]. A missense mutation at rs16891982 in a second gene, *SLC45A2*, changes the ancestral leucine to phenylalanine at amino acid 374 (*L374F*), and is 96%–98% fixed in northern Europeans [6], [7], [8]. From functional admixture studies, *SLC24A5^A111T^* and the *L374F* mutation in *SLC45A2* (*SLC45A2^L374F^*) have each been estimated to contribute about 1/3 of the difference between West African and European skin color [7]. East Asians largely lack these mutations [6], [7], [8], [9]. Contributors to European skin, hair or eye color (*ASIP* [10], *TYR* [11], *SLC24A4* [*12*], *KITLG, OCA2,* or *MC1R*, [12], [13], [14]) are not common enough among East Asians to be a primary contributor to East Asian skin color. It is reasonable to hypothesize that there exists an East Asian equivalent of *SLC24A5^A111T^*.

**Figure 1:**
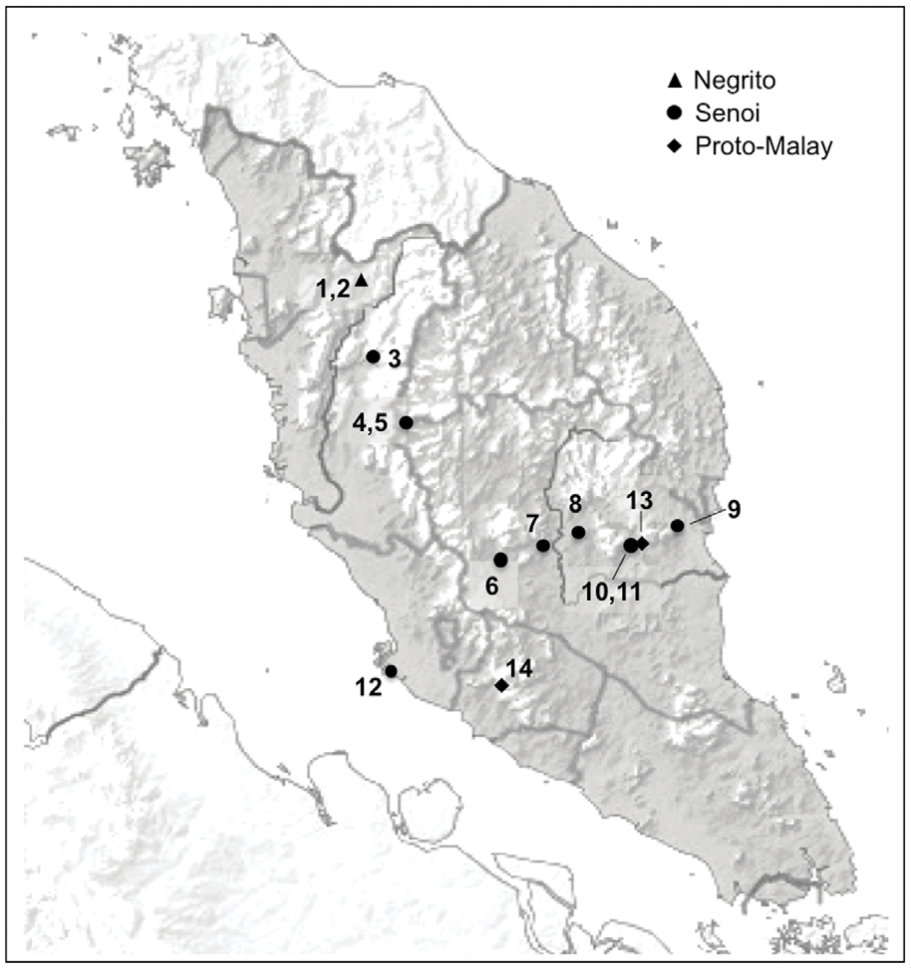
Map of Peninsular Malaysia indicating sampling sites. For Orang Asli tribes and their corresponding village locations, see Table S1.

**Table 1:** Orang Asli classification by tribe and subtribe.

		Orang Asli	
Tribe	Negrito	Senoi	Proto Malay
*Subtribe*	Batek	Che Wong*	Jakun*
	Jahai	Jah Hut*	Orang Kanak
	Kensiu*	Mah Meri*	Orang Kuala/Laut
	Kintak*	Semai*	Seletar
	Lanoh	Semak Beri*	Semelai
	Mendrik	Temiar*	Temuan*

* Denotes subtribes sampled.

We have noted that South Asians (e.g. Indians, Pakistanis, Sri Lankans) have high allele frequencies for *SLC24A5^A111T^*. Detection of the strong contributions of *SLC24A5^A111T^* to skin color among South Asians [15] has been facilitated by the scarcity of *SLC45A2^L374F^* [10] and other European skin color alleles in those populations [14]. The Senoi are believed to be an admixture between the Negrito (formerly known as Semang) and regional Mongoloid populations from Indo-China such as the Proto-Malay [16], [17]. The Senoi tribe of the Orang Asli of Peninsular Malaysia thus represents the East Asian equivalent to South Asians. In this population, putative East Asian skin color alleles would be expected to segregate with lighter skin color. Since the Senoi are expected to lack the additional mutations that are responsible for the even lighter skin of Northeastern Asians (e.g. Chinese and Japanese), using the Senoi for mapping will allow us to focus on finding the primary East Asian skin color alleles without being distracted by those additional mutations. We have characterized skin color distribution in the Orang Asli populations. We have also estimated the extent of admixture of Orang Asli populations with Europeans and South Asians by genotyping for *SLC24A5^A111T^* and *SLC45A2^L374F^*.

## Results and Discussion

### Distribution of Melanin Indices in Orang Asli

The aboriginal populations of Peninsular Malaysia, the Orang Asli (Malay for “original people”, Figure 1) are ideal for mapping the East Asian skin color genes. The Orang Asli [18] represent 0.5% of the Malaysian population, and are comprised of three indigenous tribes: the Negrito, Senoi, and Proto-Malay, and encompass 18 ethnic subtribes (Table 1) [19], [20], [21]. The Senoi are the largest in number among these tribes, followed by the Proto-Malay, and the Negrito [20], [21], [22]. The Negrito, who have dark-skin and curly hair, were the first occupants of South-East Asia and live as hunter-gatherers. The Proto-Malay, who have a lighter average skin color, straight hair, and epicanthal folds, work as farmer-traders [23], [24]. The Senoi have a wide range of skin color and wavy hair, living as both hunter-gatherers and traders, and are thought to descend from an admixture between the Negrito and an East Asian population. South Asians are lighter than West Africans but darker than Europeans, and have the commonly fixed *SLC24A5^A111T^* [15] and infrequently the *SLC45A2^L374F^* [9], [25]. Similarly, we hypothesize that the Proto-Malay and the lighter Senoi, neither of whom are as light as Northeastern Asians, share a fixed derived skin color allele.

**Table 2:** Orang Asli genotype combinations for *SLC24A5* and *SLC45A2.*

Genes and alleles	Number of Individuals	
	Homozygous	Heterozygous
*SLC24A5^A111T^*	1	39
*SLC45A2^L374F^*	1	27
Ancestral for both *SLC24A5* & *SLC45A2*	430	
*SLC24A5^A111T^* and/or *SLC45A2^L374F^*	62	
*SLC24A5^A111T^* and *SLC45A2^L374F^*	5	
**Total**	492	

The melanin index (M-index) is a measure of melanin content in skin calculated from skin reflectance [26], where higher M-indices are associated with lower reflectance and darker color. Overall, our calculated M-indices provided quantitative validation of visual impressions. A total of 517 samples were collected from the three tribes of the Orang Asli and M-indices calculated for each individual from skin reflectance measurements (Figure S1). The average M-index for all Orang Asli was 47.6. Average M-indices were highest for Negrito (55.1, *n* = 55), followed by Senoi (45.5, *n* = 412), and Proto-Malay (42.2, *n* = 50).

**Figure 2:**
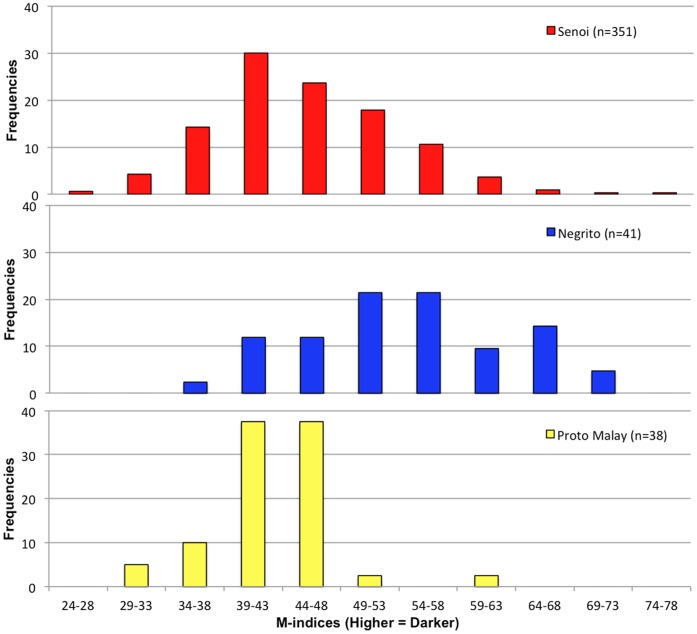
M-indices of Orang Asli who are homozygous ancestral for *SLC24A5 & SLC45A2*. Notably, the Negrito show the highest average M-index and the Proto Malay the lowest, while the Senoi have the broadest distribution of skin color. 40 Orang Asli samples containing either *SLC24A5^A111T^* or *SLC45A2^L374F^* alleles have been excluded from this plot. For plots including all individuals, see Figure S1.

To provide context for our skin color readings of the Orang Asli, we calculated average melanin indices for small samples of ethnic continental Africans, South Asians, East Asians, and Europeans. M-indices for the continental Africans ranged from 49 to 62 (average 56), within the range found for the Negrito group. M-indices for the South Asians ranged from 33 to 49 (average 40.2). M-indices for the East Asians ranged from 21 to 28 (average 25), all lighter than that of the lightest Proto-Malay (30.5). M-indices for Europeans ranged from 20 to 22 (average 21), slightly lighter than East Asians, and lighter than the lightest Orang Asli individual: a Senoi (28.1). Overall, we found the following descending order of average M-index for our tested populations: Continental Africans & Negrito > South Asians & Senoi >Proto-Malays > East Asians > Europeans. Our M-indices are consistent with those reported by Shriver et al. and Parra et al. [11], [27] and with our visual impressions.

**Figure 3:**
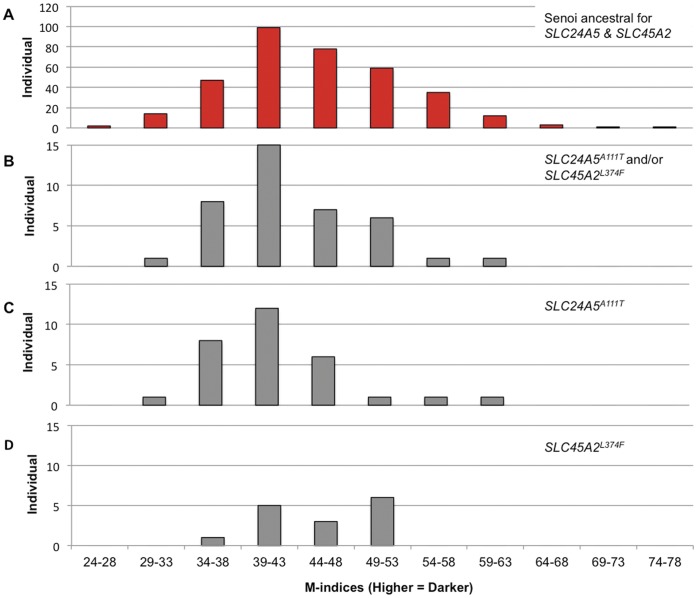
Distribution of M-indices of Senoi with and without derived alleles for *SLC24A5 and SLC45A2.* (A) Senoi samples with *SLC24A5* and *SLC45A2* ancestral alleles. (B) Senoi with derived alleles for either *SLC24A5* or *SLC45A2*, or a combination of both. (C) Senoi with the derived allele of *SLC24A5*. (D) Senoi with the derived allele of *SLC45A2*.

**Table 3:** Senoi genotype combinations for *SLC24A5* and *SLC45A2.*

Genes and alleles	Number of Individuals	
	Homozygous	Heterozygous
*SLC24A5^A111T^*	1	29
*SLC45A2^L374F^*	1	14
Ancestral for both *SLC24A5* & *SLC45A2*	351	
*SLC24A5^A111T^* and/or *SLC45A2^L374F^*	39	
*SLC24A5^A111T^* and *SLC45A2^L374F^*	5	

To confirm and quantitate our visual impression that females are of lighter skin color than males, we plotted M-indices of the Orang Asli based on gender (Figure S2). The average M-index for males was 48.28, and for females, 45.31. The females were lighter than the males by an average of 3 M-index units (ANOVA, *p*<0.001). This difference, whose genetic basis is unknown, has been hypothesized to facilitate survival by satisfying higher requirements for vitamin D associated with pregnancy and lactation [2]. Higher vitamin D levels may be necessary for normal fetal and neonatal skeletal system development [28], [29].

**Figure 4:**
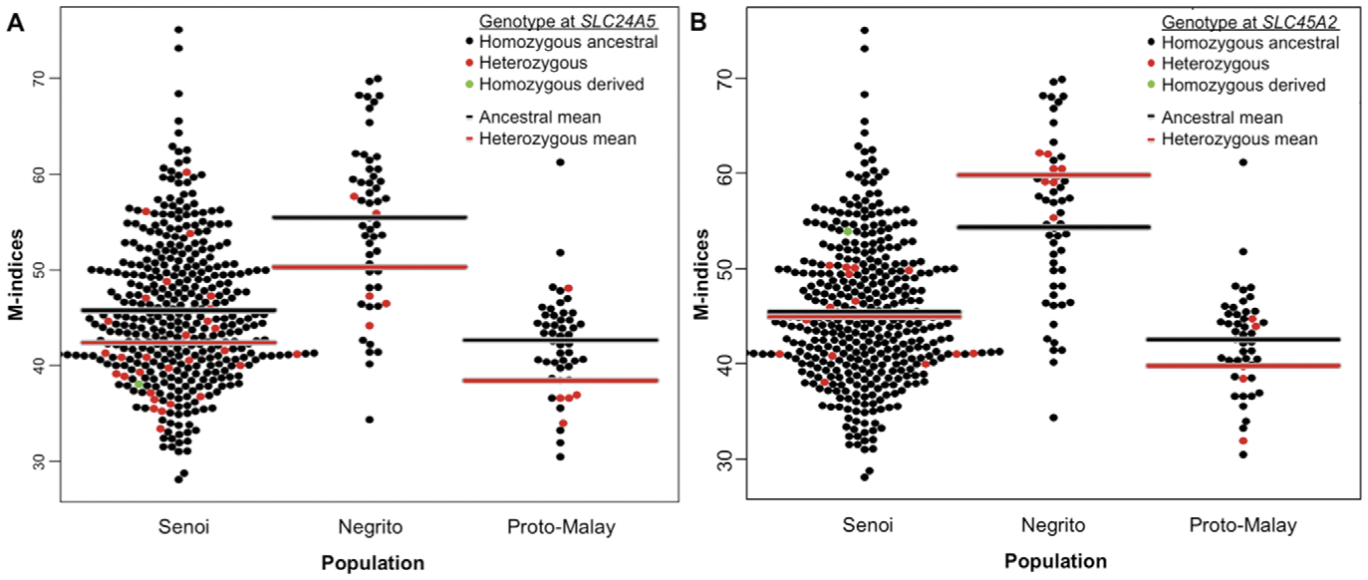
Beeswarm dotplot showing the effect of SLC24A5^A111T^ and *SLC45A2^L374F^* on the Orang Asli skin color. A) *SLC24A5^A111T^* showed a statistically significant effect on skin color (*p* = 0.0013) B) *SLC45A2^L374F^* does not reach significance effect for the Orang Asli skin color (*p* = 0.37). The black line indicates the average M-index of samples with homozygous ancestral alleles, and the red line indicates the average M-index of samples with heterozygous derived alleles.

### European Admixture in Orang Asli

Both historically and visually, European and South Asian admixtures are sporadically present in the Orang Asli. Individuals with known or strongly visual admixture were not included in the present work. European skin color alleles have the potential to interfere with the mapping of East Asian skin color. To identify specific individuals containing European skin color alleles that might interfere with mapping of East Asian skin color genes, we genotyped for the two skin color SNPs that are fixed in northern Europeans, *SLC24A5^A111T^* and *SLC45A2^L374F^.*

Among the Orang Asli, the *A111T* allele was found more frequently than *L374F*. Of 517 samples, 492 yielded a clear genotype. Of those, 438 (89.0%) were ancestral for both alleles, and 62 individuals (12.6%) had a derived allele for either *SLC24A5* or *SLC45A2* (Table 2). The full range of M-index values for the Orang Asli is covered by the 438 individuals lacking these European alleles. There were 6 individuals lighter than the lightest individual with a derived allele for either gene. This result suggests that admixture of exclusively East Asian skin color alleles, in conjunction with their ancestral alleles, can account for the full range of skin color we measured in the Orang Asli (Figure 2).

We note an excess of *SLC24A5^A111T^* over *SLC45A2^L374F^* alleles within the Orang Asli. There were 40 individuals with at least one *SLC24A5^A111T^* allele and 28 with at least one *SLC45A2^L374F^* allele. There was one homozygote for each derived allele; the individual homozygote for *SLC45A2^L374F^* was heterozygous for *SLC24A5^A111T^*. The derived allele frequencies of *SLC24A5^A111T^* and *SLC45A2^L374F^* for all the Orang Asli samples were 0.06 and 0.07, respectively; the Senoi allele frequencies were 0.04 and 0.02, respectively (Figure 3, Table 3).

Our findings provide molecular evidence that supports the possibility of admixture of the Orang Asli with South Asians and Europeans. Admixture with South Asians, who have a much higher frequency of *SLC24A5^A111T^* over *SLC45A2^L374F^* alleles [15], readily explains the allelic excess described above. Historically, the most likely origins of European admixture are British, Dutch, and Portuguese [30], who are mostly homozygous for both derived alleles [31]. Therefore, each instance of *SLC45A2^L374F^* (total 29) that is due to European admixture would be introduced along with an *SLC24A5^A111T^* allele (total 41). There were 12 (41–29) more instances of *SLC24A5^A111T^* than of *SLC45A2^L374F^*. The ratio of European to South Asian admixture can be roughly estimated from the number of Northern European *SLC45A2^L374F^* alleles (29) divided by the number of excess *SLC24A5^A111T^* alleles (12): 29/12 = 2.42. This calculation suggests Europeans as the most likely source of derived alleles at both loci. The proportion of derived alleles originating from South Asians can be estimated from the excess number of *SLC24A5^A111T^* over *SLC45A2^L374F^* alleles divided by the total number of *SLC24A5^A111T^* alleles: 12/41 = 0.29. This is a minimal estimate because varying proportions of South Asians have the *SLC45A2^L374F^* allele. Overall, about ¾ of instances of *SLC24A5^A111T^* originate from European admixture, and that the remaining ¼ can potentially be accounted for by South Asian admixture.

### Contribution of *SLC24A5^A111T^* to Skin Color in the Orang Asli

To measure the effect of each European allele on skin color in the Orang Asli as a whole, we studied the distribution of M-indices and frequency of the derived alleles (Figure 4). Linear regression was used to determine the skin color variability. For all populations sampled, *SLC24A5^A111T^* showed a significant effect on M-index (*p* = 0.0013), but the effect of *SLC45A2^L374F^* did not reach significance (*p* = 0.37). *SLC24A5^A111T^* had a significant effect on skin color in the Senoi (*p* = 0.01), but the numbers were too small reach statistical significance in the other two tribes. The population of origin and genotype explain 16.5% and 1.6% of variation in Orang Asli skin color, respectively. *SLC45A2^L374F^* showed no significant effect on skin color either in aggregate or in any one population, which may be due in part to its low frequency. Individuals carrying those two European alleles can also be expected to be more likely to carry other European mutations. Therefore, using only individuals without the *SLC45A2^L374F^* and *SLC24A5^A111T^* alleles for mapping East Asian skin color genes will diminish the likelihood of interference from European skin color alleles.

### Families in the Orang Asli Samples

Of our 351 Senoi samples with ancestral alleles for *SLC24A5^A111T^* and *SLC45A2^L374F^*, we have 9 family samples (including both parents and at least 2 children) that we can later use to correlate Mendelian inheritance of putative East Asian light skin color alleles. We predict that a child heterozygous for East Asian skin color alleles will be darker than a child homozygous for East Asian skin color alleles. In addition, we have 3 families with just 1 child, 10 families with 2 children that are missing either parent sample, and 3 families with at least 2 children and at least 1 grandchild that are missing either parent sample.

### Conclusion

We have characterized the range of skin color of the three tribes of the Orang Asli: the Negrito, the Proto-Malay, and the Senoi. The distribution of M-indices between all three tribes is consistent with visual observation: the Negrito are the darkest, the Proto-Malay are the lightest, and the Senoi (our largest population sample) exhibit the broadest distribution of skin color. We detected an average of about 11% of individuals containing either or both European skin color alleles of *SLC24A5* and *SLC45A2*. Individuals without those derived alleles covered the entire skin color spectrum of our Orang Asli samples, including even the lightest individuals. This result indicates that East Asian skin color alleles play the primary role in defining Orang Asli skin color. *SLC24A5^A111T^* had a small, statistically significant impact on Orang Asli skin color. No significant impact was detected for *SLC45A2^L374F^*. The 371 Senoi samples without either of these two confounding European alleles represent an excellent population for mapping East Asian skin color genes. This work also provides a foundation for collecting additional samples using analogous populations.

## Materials and Methods

### Ethics Statement

Written consent was obtained from each participant and this study was approved by the IRB committees of Penn State University (29269EP) and Universiti Kebangsaan Malaysia (UKM 1.5.3.5/244/FST-001-2010). We proceeded with permission from Jabatan Hal Ehwal Orang Asli, Malaysia (JHEOA) (PP.30.032 Jld 15(16)).

### Recruitment

Participants from among the Orang Asli populations listed in Table 1 were recruited with the help of JHEOA and the Malaysian Ministry of Health’s district health clinics at participating Orang Asli villages in 2010. Recruitment took place during the monthly health drive at each village. Place of birth, ancestry of parents and grandparents, and number of siblings were obtained by interview.

### Skin Reflectometry

Melanin is the major contributor to skin pigmentation [32]. Melanin content was assessed by reflectance spectrophotometry, using the L* value from the Commission International d’Eclairage (CIE) L*a*b* color system [33]. This index is based on differences in the spectral curves of hemoglobin and melanin, based on work by Diffey et al. (1984), who suggested that the reflectance of narrow-band light in the red spectrum would yield reasonable estimates of the melanin content of a person’s skin. Melanin indices were calculated using the equation:



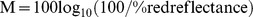
.

We measured constitutive skin pigmentation on the upper inner arm to minimize the potentially confounding effects of sun exposure and body hair. To minimize variation due to dirt and/or oil, we cleaned off each participant’s skin with cotton balls moistened with 70% ethanol. We used a Datacolor CHECK^PLUS^ spectrophotometer for our measurements, and report the calculated melanin index. Before each measurement session, the instrument was calibrated according to manufacturer instructions. To minimize blanching due to occlusion of blood from the region being measured, care was taken not to apply too much pressure to the skin [34].

### DNA Collection

5 mL of each participant’s blood was collected and mixed with an equal amount of storage buffer pH8, containing 100 mM Tris HCl, ethylenediaminetetraacetic (EDTA) acid (100 mM) and 2% sodium dodecyl sulfate [35]. Saliva samples were collected using the Oragene DNA saliva kit (DNA Genotek, Ontario, Canada). DNA from blood was extracted using phenol/chloroform or the Qiagen DNA Blood kits [#51106 & 51185]. DNA from saliva was extracted using the prepIT.L2P kit (DNA Genotek, Ontario, Canada). DNA integrity was checked by agarose gel electrophoresis and the quantity determined using a Qubit fluorometer.

### Selection of SNPs for Genotyping

SNPs rs1426654 and rs16891982 were chosen based on their known functional impact [7], and characterized by a) maximum allele frequency difference between European and East Asian populations, and b) genomic regions of diminished variation in European populations (CHB/JPT) of about 150 and 40 kb, respectively. SNP chips were customized through Applied Biosystems TaqMan Arrays, and genotyping was performed at Huck Institute of Life Sciences, Genomics Core Facility, University Park, PA and at Penn State College of Medicine, Hershey, PA.

## Supporting Information

Figure S1 M-indices of all sampled Orang Asli with clear genotype. This plot includes individuals with either ancestral or derived alleles for *SLC24A2* and *SLC45A2* (*n* = 492).
(TIF)

Figure S2 Distribution of Orang Asli M-indices based on sex for all Orang Asli samples. The average M-index of males was darker than that of females by 3 M-index units (*n = *517, ANOVA, *p*<0.0001, R^2^ = 3.2%). This plot includes individuals whose DNA did not yield clear genotype for alleles at *SLC24A5* or *SLC45A2*.
(TIF)

Table S1 Locations of sampling villages and their tribe and subtribe(s).
(DOCX)
