# Correction: Skin Color Variation in Orang Asli Tribes of Peninsular Malaysia

**DOI:** 10.1371/annotation/dfb89198-58fe-49e5-be23-325be58fedd0

**Published:** 2012-09-11

**Authors:** Khai C. Ang, Mee S. Ngu, Katherine P. Reid, Mei S. Teh, Zamzuraida S. Aida, Danny XR. Koh, Arthur Berg, Stephen Oppenheimer, Hood Salleh, Mahani M. Clyde, Badrul M. Md-Zain, Victor A. Canfield, Keith C. Cheng

Some formatting errors occurred in Tables 1, 2, and 3. Please review the correctly formatted tables here:

Table 1: 

**Figure:**
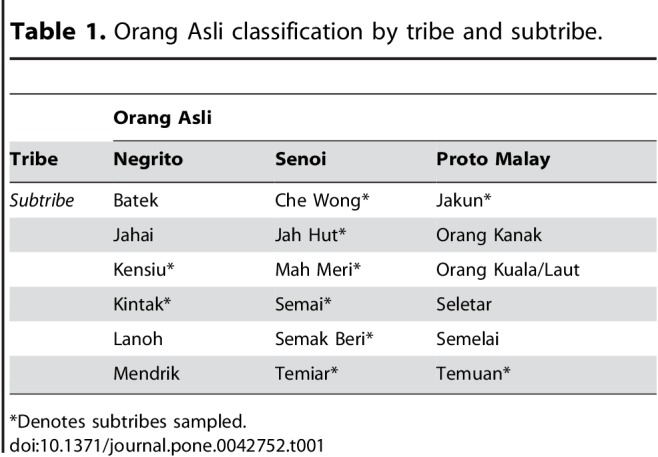


[^]

Table 2: 

**Figure:**
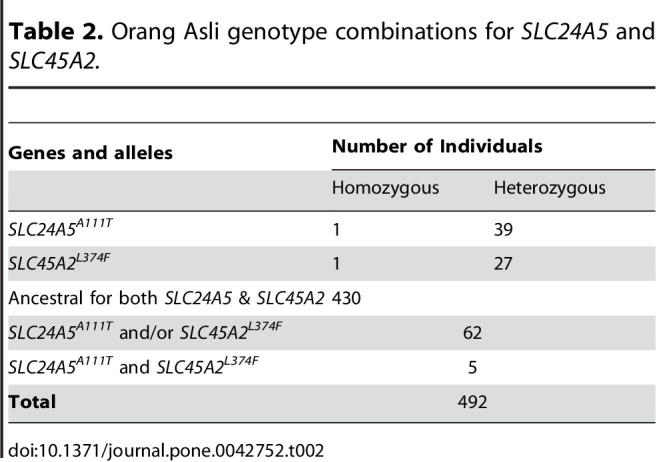


[^]

Table 3: 

**Figure:**
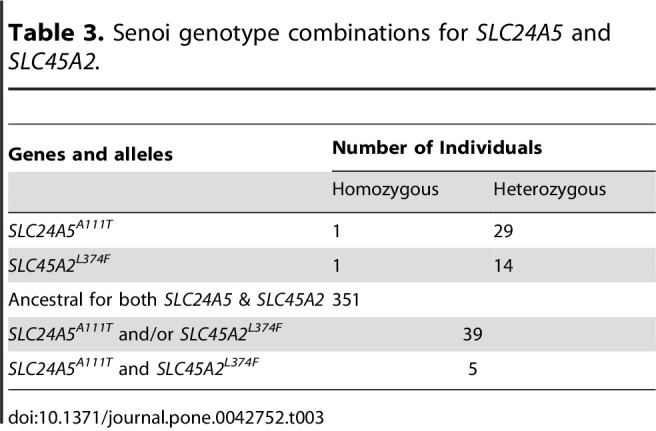


[^] 

