# Supplementary figures and images for: Skin Color Variation in Orang Asli Tribes of Peninsular Malaysia
Source: PLoS One. 2012 Aug 13;7(8):e42752. doi: 10.1371/journal.pone.0042752 (PMC3418284; doi:10.1371/journal.pone.0042752)

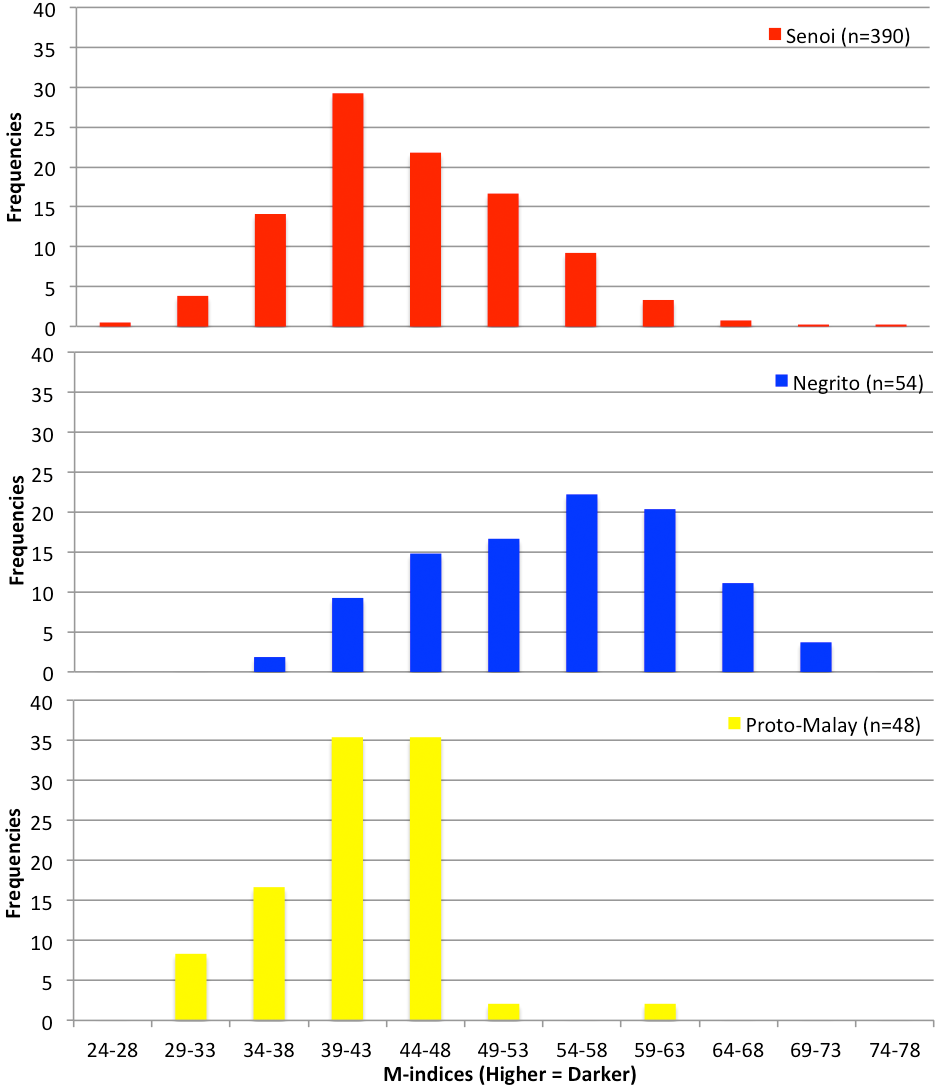

Supplement: Figure S1 — M-indices of all sampled Orang Asli with clear genotype. This plot includes individuals with either ancestral or derived alleles for SLC24A2 and SLC45A2 (n = 492). (TIF) [file pone.0042752.s001.tif]

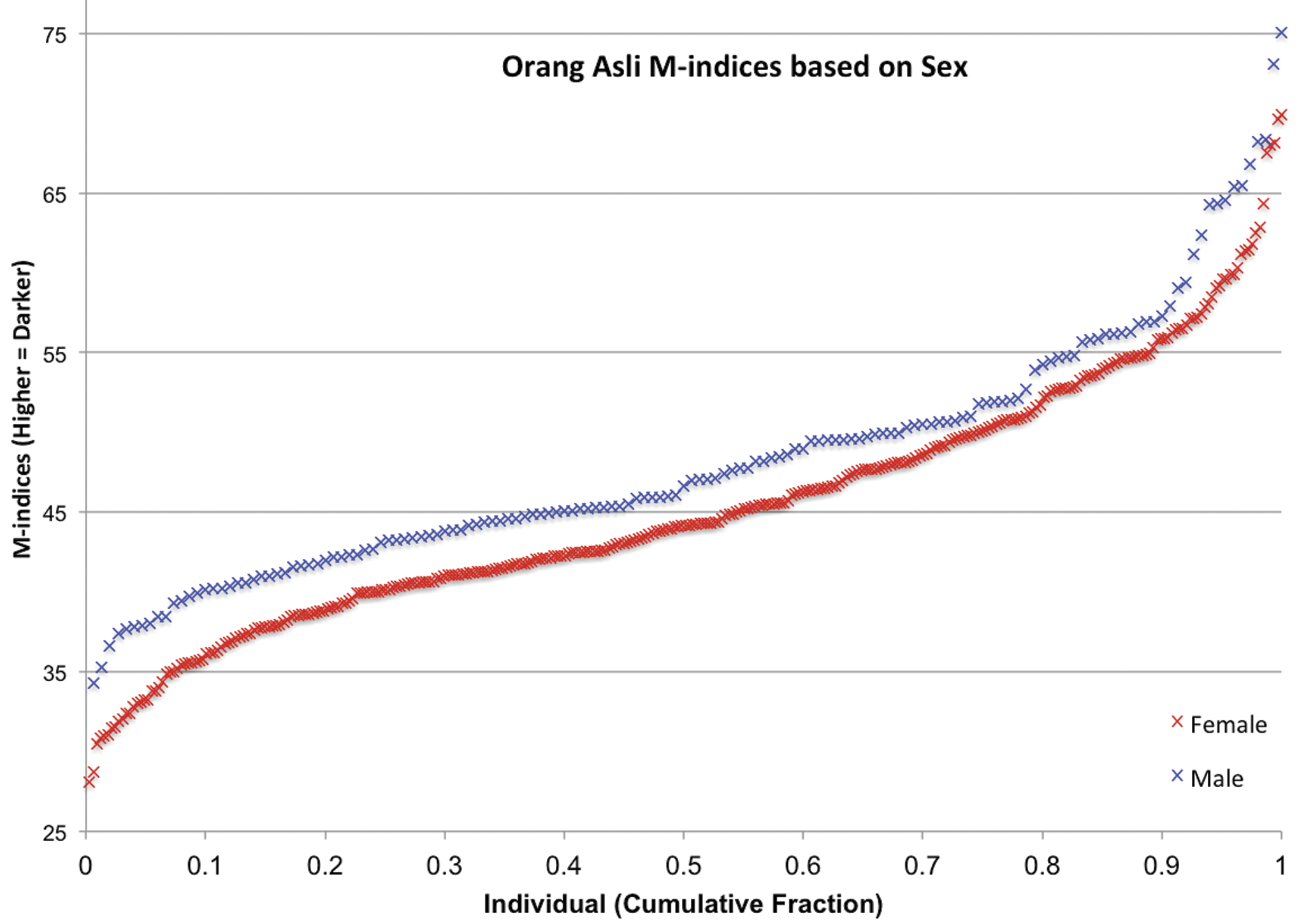

Supplement: Figure S2 — Distribution of Orang Asli M-indices based on sex for all Orang Asli samples. The average M-index of males was darker than that of females by 3 M-index units (n = 517, ANOVA, p<0.0001, R2 = 3.2%). This plot includes individuals whose DNA did not yield clear genotype for alleles at SLC24A5 or SLC45A2. (TIF) [file pone.0042752.s002.tif]
