# Supplementary material for: Skin Color Variation in Orang Asli Tribes of Peninsular Malaysia
Source: PLoS One. 2012 Aug 13;7(8):e42752. doi: 10.1371/journal.pone.0042752 (PMC3418284; doi:10.1371/journal.pone.0042752)
Supplement: Table S1 — Locations of sampling villages and their tribe and subtribe(s). (DOCX) [file pone.0042752.s003.docx]

**Table S1:** **Locations of sampling villages and their tribe and subtribe(s).**

| Number | Tribe | Subtribe | Village |
| --- | --- | --- | --- |
| 1 | Negrito | Kintak | Bukit Asu, Baling |
| 2 | Negrito | Kensiu | Lubuk Legong, Baling |
| 3 | Senoi | Temiar | Kg Kenang, Sg. Siput |
| 4 | Senoi | Semai | Kg Boh, Cameron Highlands |
| 5 | Senoi | Semai | Sg Ruil, Cameron Highlands |
| 6 | Senoi | Che Wong | Sg. Enggang, Lanchang |
| 7 | Senoi | Semak Beri | Paya Sendayan, Temerloh |
| 8 | Senoi | Jah Hut | Kg. Paya Reko, Kuala Krau |
| 9 | Senoi | Semak Beri & Jakun | Sg. Emas, Sg. Lembing |
| 10 | Senoi | Semak Beri | Batu 55, Maran |
| 11 | Senoi | Semak Beri | Kampung Merbau, Maran |
| 12 | Senoi | Mah Meri | Sg. Bumbun, Pulau Carey |
| 13 | Proto-Malay | Jakun | Seri Makmur, Maran |
| 14 | Proto-Malay | Temuan | Kg. Tekir, Nilai |

*Arranged by tribes and then, geographically from the North to the South of Peninsular Malaysia.
